# Fish Scale Collagen Peptides Protect against CoCl_2_/TNF-*α*-Induced Cytotoxicity and Inflammation via Inhibition of ROS, MAPK, and NF-*κ*B Pathways in HaCaT Cells

**DOI:** 10.1155/2017/9703609

**Published:** 2017-06-22

**Authors:** Fazli Subhan, Hae Yeong Kang, Yeseon Lim, Muhammad Ikram, Sun-Yong Baek, Songwan Jin, Young Hun Jeong, Jong Young Kwak, Sik Yoon

**Affiliations:** ^1^Department of Anatomy, Pusan National University School of Medicine, Yangsan, Gyeongsangnam-do 50612, Republic of Korea; ^2^Pioneer Research Center, Suwon 16499, Republic of Korea; ^3^Department of Mechanical Engineering, Korea Polytechnic University, Siheung 15073, Republic of Korea; ^4^Kyungpook National University, Daegu 41566, Republic of Korea; ^5^Department of Pharmacology, Ajou University School of Medicine, Suwon 16499, Republic of Korea

## Abstract

Skin diseases associated with inflammation or oxidative stress represent the most common problem in dermatology. The present study demonstrates that fish scale collagen peptides (FSCP) protect against CoCl_2_-induced cytotoxicity and TNF-*α*-induced inflammatory responses in human HaCaT keratinocyte cells. Our study is the first to report that FSCP increase cell viability and ameliorate oxidative injury in HaCaT cells through mechanisms mediated by the downregulation of key proinflammatory cytokines, namely, TNF-*α*, IL-1*β*, IL-8, and iNOS. FSCP also prevent cell apoptosis by repressing Bax expression, caspase-3 activity, and cytochrome c release and by upregulating Bcl-2 protein levels in CoCl_2_- or TNF-*α*-stimulated HaCaT cells. In addition, the inhibitory effects of FSCP on cytotoxicity and the induction of proinflammatory cytokine expression were found to be associated with suppression of the ROS, MAPK (p38/MAPK, ERK, and JNK), and NF-*κ*B signaling pathways. Taken together, our data suggest that FSCP are useful as immunomodulatory agents in inflammatory or immune-mediated skin diseases. Furthermore, our results provide new insights into the potential therapeutic use of FSCP in the prevention and treatment of various oxidative- or inflammatory stress-related inflammation and injuries.

## 1. Introduction

The epidermis, the outermost layer of the skin, plays a pivotal role in the skin barrier to protect the body from causative environmental agents of skin injury, such as excessive water loss, pathogenic microorganisms, allergens, physical or chemical irritants, and radiation [1]. The epidermis is an avascular stratified epithelium that obtains nourishment from blood vessels in the dermis of the skin. Keratinocytes are the predominant cell type in the epidermis and are primarily involved in the formation of the skin barrier. Moreover, keratinocytes play an important role in skin immunity [2, 3]. Oxidative stress is known to damage the skin and is a key factor in the development of diverse clinical conditions, such as skin aging, inflammatory skin diseases, and skin cancer [4]. Oxidative stress results from an imbalance between reactive oxygen species (ROS) and antioxidant defense mechanisms that neutralize ROS. Ultraviolet radiation from the sun is one of the most common stimulators of ROS production, which can damage DNA, cellular proteins/lipids, and cell membranes, and induce skin inflammation, which can ultimately lead to apoptotic or necrotic cell death [5]. In addition to oxidative stress, various other harmful stimuli, such as trauma, pathogens, irritants, allergens, damaged cells, and cytokines such as tumor necrosis factor-*α* (TNF-*α*), can induce keratinocyte activation. In turn, activation of keratinocytes elicits an inflammatory response, leading to the release of inflammatory and immunomodulatory mediators that include TNF-*α*, interleukin-1*β* (IL-1*β*), IL-8, and inducible nitric oxide synthase (iNOS) [6]. These mediators disrupt the skin barrier and recruit and trap inflammatory and immune cells, such as monocytes/macrophages, neutrophils, mast cells, NK cells, eosinophils, and T-cells in inflammatory lesions in the skin [7, 8]. In particular, TNF-*α*, which is produced by multiple cell types in the skin, including keratinocytes, plays a key role in inflammatory and immune cell recruitment and activation [9]. TNF-*α* stimulates the production of inflammatory and immunomodulatory mediators by keratinocytes, which in turn activates mitogen-activated protein kinase (MAPK) and nuclear factor-*κ*B (NF-*κ*B) signal transduction pathways, ultimately leading to cell injury and apoptotic death [6].

Hypoxic injury of the skin is an important pathophysiological mechanism underlying several common clinical conditions, such as pressure ulcer, diabetic ulcer, and venous ulcer [10]. Hypoxia-induced injury of skin is known to result in oxidative stress and inflammatory responses [11]. Inflammation is the body's way of protecting itself against harmful stimuli. However, excessive inflammation can cause severe tissue damage, which can in turn contribute to the development of diseases such as rheumatoid arthritis, diabetes, atherosclerosis, and cancer [12]. Therefore, inhibiting the production of inflammatory mediators can serve as a potential therapeutic approach for the treatment of various inflammatory diseases. Identification of effective anti-inflammatory agents to abrogate the generation of important inflammatory mediators from damaged keratinocytes is a critical first step in the development of therapeutic drug candidates against inflammatory skin diseases.

Recently, human umbilical vein endothelial cells (HUVECs) cultured on collagen derived from fish scales or bullfrog skin were found to exhibit enhanced cell attachment and proliferation, accompanied by reduced cell surface expression of ICAM-1 and VCAM-1, markers of endothelial cell activation and inflammation [13]. In addition, the fish scale collagen peptides (FSCP) were observed to promote HUVEC proliferation and reduce IL-6, IL-8, and TNF-*α* production in lipopolysaccharide-stimulated HUVECs [14]. Currently, FSCP have increasingly gained attention because of their safety [15], and diverse biological properties, including antioxidant [16], antitumor [17], antihypertensive [18], neuroprotective [17], antiskin aging [19], epiphyseal growth-promoting [20] and wound healing [21] effects, and osteogenic/endothelial differentiation-promoting effects in rat bone marrow mesenchymal stem cells [22]. Furthermore, oral administration of FSCP was shown to diminish the production of proinflammatory cytokines such as TNF-*α* and NO in rat synoviocytes and NO and C-reactive protein in diabetic patients with chronic inflammation [23]. However, whether FSCP can modulate the biological activity and inflammatory responses of keratinocytes remains to be explored.

Cobalt chloride (CoCl_2_), a hypoxia-mimetic agent, is known to induce ROS generation and cytotoxicity [24]. CoCl_2_ exposure triggers the inflammatory response and is associated with enhanced secretion of IL-6 and IL-8 in human epithelial and endothelial cells [25]. Thus, in the present study, we investigated the biological behavior and the modulatory effects of FSCP on inflammation in HaCaT cells, a widely recognized model skin keratinocyte cell line that is commonly used in dermatopathological studies. In particular, cytotoxicity and inflammatory responses were elicited by treating HaCaT cells with CoCl_2_ and TNF-*α*, respectively. Here, we report that FSCP exert protective effects against CoCl_2_/proinflammatory cytokine-induced injuries and inflammatory responses in HaCaT cells by inhibiting the ROS, MAPK, and NF-*κ*B signal transduction pathways.

## 2. Methods

### 2.1. Cell Culture and Reagents

HaCaT cells, spontaneously immortalized human epidermal keratinocytes, were purchased from CLS Cell Line Service (Eppelheim, Germany) and cultured in Dulbecco's modified Eagle's medium (Hyclone, GE Healthcare Life Sciences, Logan, UT, USA) supplemented with 10% fetal bovine serum, 100 IU/mL penicillin, and 100 mg/mL streptomycin (all from Gibco, Thermo Fisher Scientific, Carlsbad, CA, USA) in a humidified 5% CO_2_ atmosphere at 37°C. Subconfluent cells were harvested with trypsin-EDTA and used for further experiments. Culture media were replaced every three days.

CoCl_2_, 2′,7′-dichlorodihydrofluorescein diacetate (DCFH-DA), N-acetyl-L-cysteine (NAC), 4′,6-diamidino-2-phenylindole (DAPI), and bicinchoninic acid were purchased from Sigma-Aldrich (St. Louis, MO, USA). Human recombinant TNF-*α* was provided by PeproTech (Rocky Hill, NJ, USA). Antibodies against ERK, phospho-ERK (p-ERK), JNK, phospho-JNK (p-JNK), p38/MAPK, phospho-p38/MAPK (p-p38/MAPK), Bax, caspase-3, and cleaved caspase-3 were supplied by Cell Signaling (Cambridge, MA, USA). An antibody against iNOS (AHP303) was purchased from Serotec (Oxford, UK). Antibodies against Bcl-2 and NF-*κ*B p65 were obtained from Santa Cruz Biotechnology (Santa Cruz, CA, USA). The p-p38/MAPK inhibitor SB203580 and FSCP extracted from tilapia were provided by Tocris Cookson (Ellisville, MO, USA) and Geltech (Busan, Korea), respectively. All other reagents and compounds used were supplied by Sigma-Aldrich.

### 2.2. Mass Spectrometry (MS) Analysis of FSCP

The molecular weight distribution of FSCP was analyzed using a TripleTOF 4600 LC/MS/MS system (AB SCIEX, Framingham, MA, USA). Samples were directly injected into the mass spectrometer through an electrospray ionization source operated in the positive ion mode. A syringe pump was used to inject samples at the flow rate of 20 *μ*L/min. MS data were collected over *m*/*z* values ranging from 100 to 3000. PeakView analysis software (AB SCIEX) was used for data analysis. A mass spectrum for each sample was generated based on the total ion chromatogram (sum of all ion counts) from *m*/*z* values between 100 and 3000. A peak list having *m*/*z* values for all peaks with their abundances was generated from the mass spectrum.

### 2.3. Cell Viability Assay

HaCaT cells (1 × 10^4^ cells/well) in 96-well flat-bottom culture plates (SPL Life Sciences, Pocheon, Korea) were treated with indicated doses of FSCP for 24 h with or without CoCl_2_. Cell viability was determined using the colorimetric WST-1 conversion assay (EZ-Cytox assay kit, Daeil Lab Service, Seoul, Korea). WST-1 reagent (10 *μ*L) was added to each well, after which cells were incubated for 2 h in a humidified incubator at 37°C under 5% CO_2_. Absorbance of the formazan dye, generated by the reaction between dehydrogenase and WST-1 in metabolically active cells, was measured using a microplate reader (Tecan, Männedorf, Switzerland) at 450 nm according to the manufacturer's instructions. Percent cell viability was calculated. Experiments were performed at least thrice.

### 2.4. ROS Measurement

The effects of FSCP on ROS production in CoCl_2_- or TNF-*α*-treated HaCaT cells were determined using DCFH-DA, a ROS-sensitive fluorescent probe, under a fluorescent microscope. Cell-permeable DCFH-DA is a nonfluorescent dye that is converted to the highly fluorescent 2′7′-dichlorodihydrofluorescein (DCF) when oxidized by ROS. HaCaT cells (1 × 10^5^ cells/well) in six-well culture plates were either cotreated with CoCl_2_ (100 *μ*M) and 0.1% FSCP for 24 h or treated with 0.1% FSCP for 12 and 24 h before treatment with TNF-*α* (20 ng/mL) for 12 h. After removing the culture medium, cells were washed with phosphate-buffered saline (PBS) and incubated in 10 *μ*M DCFH-DA in fresh serum-free medium for 30 to 40 min in a humidified incubator at 37°C with 5% CO_2_ under dark conditions. Labeled cells were observed using an epifluorescence microscope (BX50, Olympus, Tokyo, Japan) or analyzed by FACSCanto II (BD Biosciences, San Jose, CA, USA). Photomicrographs were digitally acquired at 1360 × 1024 pixel resolution with an Olympus DP70 digital camera.

### 2.5. RNA Isolation and Reverse Transcriptase-Polymerase Chain Reaction (RT-PCR)

To determine gene expression levels, HaCaT cells (1 × 10^5^ cells/well) in six-well culture plates were either cotreated with CoCl_2_ (100 *μ*M) and different doses of FSCP (0.005, 0.01, 0.02, 0.5, or 0.1%) for 24 h or treated with 0.1% FSCP for 3, 12, and 24 h before treatment with TNF-*α* (20 ng/mL) for 12 h. Total RNA was extracted from the cells using Trizol reagent (Invitrogen, Carlsbad, CA, USA) following the manufacturer's instructions. RNA quantity and quality were assessed using a Nanodrop 2000 (Thermo Fisher Scientific, Waltham, MA, USA). Briefly, samples were transferred to a tube containing 1 mL of the RNA extraction solution. The homogenate was then chloroform-extracted, precipitated with isopropanol, washed with ethanol, and resuspended in 30 *μ*L of distilled water. RNA concentration and purity were determined by measuring absorbance at 260 and 280 nm. Samples had 260/280 absorbance ratios greater than or equal to 1.9. First-strand cDNA synthesis was performed by reverse transcription using 2 *μ*g of total RNA. The reaction was conducted in a 25 *μ*L reaction mixture containing 0.5 *μ*g oligo (dT) 12–18 primers (Promega, Madison, WI, USA), 50 mM Tris-HCl (pH 8.3), 75 mM KCl, 3 mM MgCl_2_, 40 mM dithiothreitol, 0.5 mM deoxynucleotide triphosphate mixture (Promega), 10 U RNase inhibitor (Promega), and 200 U of Moloney murine leukemia virus reverse transcriptase (Promega). The mixture was incubated at 37°C for 60 min, and the reaction was terminated by heating at 70°C for 5 min. The obtained cDNA was used as a template for PCR amplification using the gene-specific primers. Primer sequences are shown in Table 1. PCR amplification of cDNA was performed in an automated thermal cycler (PC-320, Astec, Osaka, Japan) in a final reaction volume of 25 *μ*L containing the following components: 4 *μ*L of cDNA solution, 20 mM Tris-HCl (pH 8.4), 50 mM KCl, 1.5 mM MgCl_2_, 0.1% Triton X-100, 0.2 mM deoxynucleotide triphosphate mixture (Promega), 0.5 pmol of each primer, and 5 U of Taq DNA polymerase (Promega). Amplified products were analyzed by electrophoresis in a 2% agarose gel and visualized by ethidium bromide staining under ultraviolet light. Band intensities of PCR products were measured using an image analysis program (MetaMorph, Universal Imaging Corporation, Downingtown, PA, USA). Results were expressed as ratios versus GAPDH mRNA amplified from same cDNA samples.

### 2.6. Total Protein Extraction and Western Blot Analysis

To determine protein expression levels, HaCaT cells (1 × 10^6^ cells/dish) that reached 70 to 80% confluence in 100 mm culture dishes (SPL Life sciences) were either cotreated with CoCl_2_ (100 *μ*M) and 0.1% FSCP for 24 h or treated with 0.1% FSCP for 12 and 24 h, followed by treatment with TNF-*α* (20 ng/mL) for 12 h. Cells from each treatment group were harvested and washed twice in cold Tris-buffered saline (TBS). Western blotting was performed by lysing the cells in 20 mM Tris-HCl buffer (pH 7.4) containing a protease inhibitor mixture (0.1 mM phenylmethanesulfonyl fluoride, 5 mg/mL aprotinin, 5 mg/mL pepstatin A, and 1 mg/mL chymostatin). Equal amounts of protein samples were heated for 10 min at 95°C in sample buffer and separated by 10 or 12% sodium dodecyl sulfate-polyacrylamide gel electrophoresis using a Mini-Protean III system (Bio-Rad, Hercules, CA, USA). Proteins were transferred onto a polyvinylidene difluoride membrane (Bio-Rad) via semidry transfer (Bio-Rad). The membrane was incubated overnight at 4°C with anti-ERK, anti-p-ERK, anti-JNK, anti-p-JNK, anti-p38/MAPK, anti-p-p38/MAPK, anti-Bax, anti-caspase-3, anticleaved caspase-3, anti-iNOS, anti-Bcl-2, and anti-NF-*κ*B p65 antibodies at 1 : 500 or 1 : 1000 dilution in TBS (20 mM Tris-HCl, 150 mM NaCl, pH 7.4) containing 3% bovine serum albumin (BSA). After washing thrice with TBS-T (TBS containing 0.1% Tween 20), the membrane was incubated for 2 h at room temperature with secondary antibodies, namely, goat anti-rabbit IgG HRP conjugate (sc-2004, Santa Cruz Biotechnology) and rabbit anti-goat IgG HRP conjugate (sc-2768, Santa Cruz Biotechnology) at 1 : 10,000 dilution and subsequently washed thrice with TBS-T. Immunoreactivity was detected with enhanced chemiluminescence (ECL, Super Signal West Pico Chemiluminescent Substrate kit, Pierce, Rockford, IL, USA) following the manufacturer's instructions. Images were acquired and quantified using a LAS-3000 imaging system (Fujifilm, Tokyo, Japan).

### 2.7. Preparation of Cytosolic and Nuclear Cell Extracts

HaCaT cells (1 × 10^6^ cells/dish) that have reached 70 to 80% confluency in 100 mm culture dishes (SPL Life Sciences) were washed with cold PBS and resuspended in 70 *μ*L of buffer A [10 mM HEPES (pH 7.9), 1.5 mM MgCl_2_, 10 mM KCl, 0.5 mM DTT, 0.5 mM PMSF, and Protease Inhibitor Cocktail (Sigma)]. Cells were incubated on ice for 15 min, after which the mixture was added with 0.5% Nonident P-40 and vortexed for 10 s to lyse the cells. Next, cells were centrifuged at 6500 rpm for 60 s at 4°C to obtain the cytosolic cell extracts. Nuclei were resuspended in 50 *μ*L of buffer C [20 mM HEPES (pH 7.9), 1.5 mM MgCl_2_, 420 mM NaCl, 0.2 mM EDTA, 25% *v*/*v* glycerol, 0.5 mM PMSF, and Protease Inhibitor Cocktail (Sigma)] and incubated on ice for 20 min with gentle pipetting every 5 min. Nuclear cell extracts were recovered after centrifugation for 10 min at 12000 rpm at 4°C. Protein concentration was determined using Bradford protein assay reagent (Bio-Rad).

### 2.8. Immunofluorescence Analysis

To detect translocation of the NF-*κ*B p65 subunit, HaCaT cells (1 × 10^5^ cells/dish) reaching 70 to 80% confluency in 60 mm culture dishes (SPL Life Sciences) were treated with 0.1% FSCP for 12 and 24 h before treatment with TNF-*α* (20 ng/mL) for 12 h. For confocal laser scanning microscopic analysis, cultured cells were washed with PBS and fixed with cold 4% paraformaldehyde in 0.1 M phosphate buffer for 20 min. The fixative was then removed by washing the cells thrice for 5 min with cold PBS, followed by permeabilization with 0.1% Triton X-100 in PBS for 5 min. After washing with cold PBS, cells were incubated with 1% BSA (Sigma-Aldrich) for 1 h at room temperature to reduce nonspecific binding. After blocking, cells were incubated with mouse anti-NF-*κ*B p65 antibody (diluted 1 : 50) at 4°C overnight. Cells were washed with PBS thrice, each for 3 min. Next, cells were incubated with FITC-conjugated anti-mouse antibody (Santa Cruz, diluted 1 : 50) for 2 h at room temperature, rinsed in cold PBS, and mounted on glass slides using Vectashield® containing DAPI (Vector Laboratories, Burlingame, CA, USA). Cell fluorescence was observed using a confocal laser scanning microscope (Olympus).

### 2.9. Statistical Analysis

Results were expressed as means ± SD for each condition. Analysis was performed using two-tailed Student's *t*-test. Statistically significant differences between the untreated control group and the CoCl_2_/TNF-*α*-treated group were considered at ^#^*p* < 0.05, and differences between the CoCl_2_/TNF-*α*-treated group and the combined FSCP and CoCl_2_/TNF-*α* cotreated group were considered at ^∗^*p* < 0.05, ^∗∗^*p* < 0.01, and ^∗∗∗^*p* < 0.001.

## 3. Results

### 3.1. FSCP Characterization

As shown in Supplementary Figure 1 available online at https://doi.org/10.1155/2017/9703609, the molecular weights of FSCP ranged from 0.1 to 1.3 kDa. The amino acid composition of FSCP, provided by the manufacturer, is shown in Supplementary Table 1. Glycine is the most abundant amino acid in FSCP, constituting 24.4 to 26.7% of total amino acid residues, followed by proline, alanine, hydroxyproline, arginine, glutamic acid, and aspartic acid residues, which range from 9.3 to 13.3%, 9.1 to 12.0%, 7.3 to 7.7%, 5.7 to 9.3%, 5.4 to 8.9%, and 4.6 to 5.5%, respectively. No other amino acids are represented by more than 5% (Supplementary Table 1).

### 3.2. FSCP Promote Cell Proliferation and Inhibit CoCl_2_-Induced Cytotoxicity

**Figure 1:**
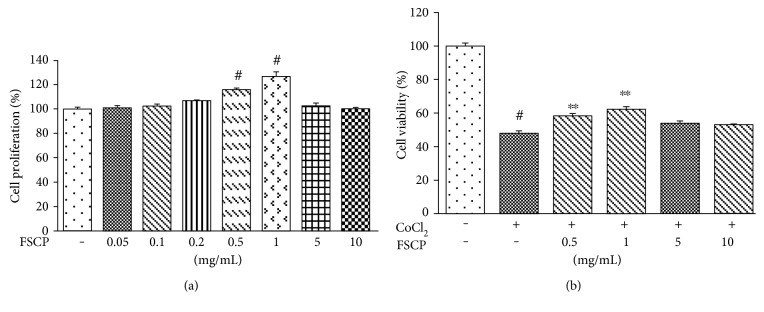
Stimulatory effects of FSCP on proliferation and viability of HaCaT cells. Treatment of HaCaT cells with FSCP for 24 h significantly enhanced cell proliferation (a) and attenuated the CoCl_2_-induced (500 *μ*M CoCl_2_) decrease in cell viability (b). Results are presented as the means ± SD of three independent experiments. ^#^*p* < 0.05 versus the control and ^∗∗^*p* < 0.01 versus the CoCl_2_-treated group.

A WST-1-based colorimetric cell proliferation assay was employed to evaluate the ability of FSCP to facilitate cell proliferation. Treatment of HaCaT cells with 0.5 to 1 mg/mL FSCP for 24 h significantly enhanced cell proliferation by 15.8 ± 0.2 and 26.7 ± 1.0% compared to the control, respectively (Figure 1(a)). In addition, we investigated the effects of 0.5 to 1 mg/mL FSCP on CoCl_2_-induced cytotoxicity of HaCaT cells using the WST-1 assay. As shown in Figure 1(b), exposure of HaCaT cells to 500 *μ*M CoCl_2_ for 24 h resulted in a 52.0 ± 1.9% decrease in cell viability compared to the control. However, decreased cell viability due to 500 *μ*M CoCl_2_ treatment was significantly ameliorated by treatment with 0.5 and 1 mg/mL FSCP for 24 h by 80.1 ± 1.9 and 72.5 ± 1.9%, respectively. Thus, the results indicate that 0.5 to 1 mg/mL FSCP confer protective effects against CoCl_2_-induced cytotoxicity in HaCaT cells (Figure 1(b).

### 3.3. FSCP Attenuate CoCl_2_/TNF-*α*-Induced ROS Generation

**Figure 2:**
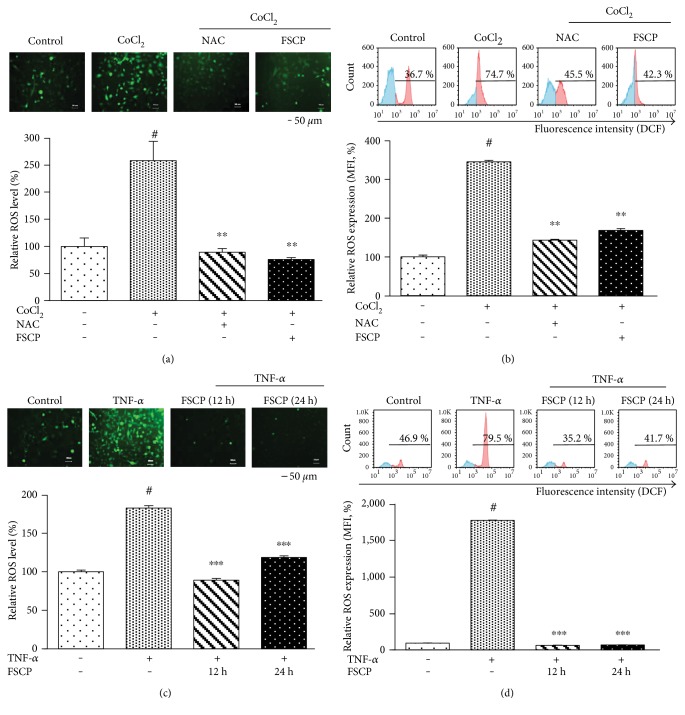
Inhibitory effects of FSCP on CoCl_2_/TNF-*α*-induced ROS generation in HaCaT cells. Intracellular ROS levels were determined via fluorescence microscopy (a, c) or flow cytometry (b, d) using DCFH-DA. The increased ROS levels induced by 100 *μ*M CoCl_2_ treatment was significantly attenuated by treatment with 1 mg/mL FSCP for 24 h or NAC for 2 h (a, b). Elevated ROS levels were induced by treatment with 20 ng/mL TNF-*α* but were reversed by treatment with 1 mg/mL FSCP for 12 or 24 h (c, d). Staining intensities were measured using ImageJ software (a, c). Blue histograms represent ROS negative cells, red histograms represent ROS positive cells, and the median fluorescence intensity (MFI) of ROS expression is plotted along the *y*-axes (b, d). Results are presented as the means ± SD of three independent experiments. ^#^*p* < 0.05 versus the control and ^∗∗^*p* < 0.01 and ^∗∗∗^*p* < 0.001 versus the CoCl_2_/TNF-*α*-treated group.

To determine whether the cytoprotective effects of FSCP are mediated by antioxidant mechanisms in CoCl_2_/TNF-*α*-stimulated HaCaT cells, intracellular ROS levels were measured via fluorescence microscopy and flow cytometry using DCFH-DA. A significant increase in ROS levels was observed after exposure of HaCaT cells to 100 *μ*M CoCl_2_ for 24 h (Figures 2(a) and 2(b)) or 20 ng/mL TNF-*α* for 12 h (Figures 2(c) and 2(d)). The pronounced increase in ROS levels as a result of 100 *μ*M CoCl_2_ treatment declined to reach near control levels upon treatment with FSCP (1 mg/mL) for 24 h compared to the CoCl_2_-treated group as demonstrated by fluorescence microscopy and flow cytometry (Figures 2(a) and 2(b)). The data obtained by fluorescence microscopic analysis coincide fairly well with those from flow cytometric analysis. Furthermore, treatment with FSCP (1 mg/mL) for 12 or 24 h significantly reversed the TNF-*α*-induced increase in ROS levels to near control values, compared to the TNF-*α*-treated group as detected by fluorescence microscopy (Figure 2(c)). This result was also confirmed by the flow cytometric analysis (Figure 2(d)). These results indicate that FSCP can restore the endogenous antioxidant defense mechanisms impaired by CoCl_2_ or TNF-*α* (Figures 2(a)–2(d)). Furthermore, NAC, a common ROS scavenger, markedly reduced 100 *μ*M CoCl_2_-induced increase in ROS levels in HaCaT cells close to normal control levels compared to CoCl_2_-treated group (Figures 2(a) and 2(b)). The above findings indicate that the inhibition of CoCl_2_-induced cytotoxicity can be attributed to the antioxidant effects of FSCP in HaCaT cells.

### 3.4. FSCP Suppress CoCl_2_-Induced Expression of Proinflammatory Cytokines

**Figure 3:**
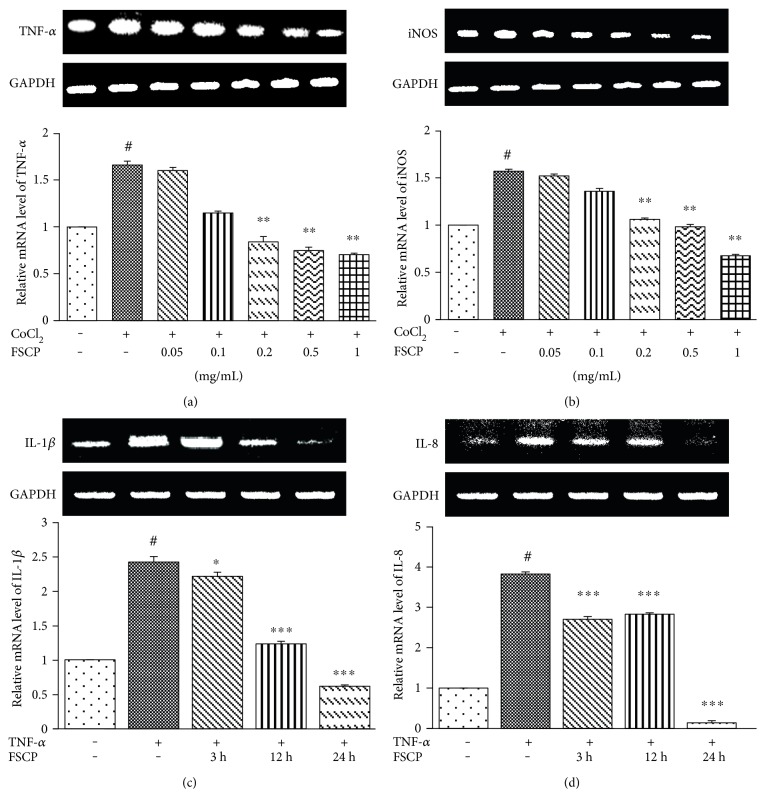
Inhibitory effects of FSCP on CoCl_2_/TNF-*α*-induced proinflammatory cytokine expression. The expression of the key proinflammatory cytokines, namely, TNF-*α*, IL-1*β*, IL-8, and iNOS, was analyzed via RT-PCR. Elevated levels of TNF-*α* and iNOS mRNA in CoCl_2_-treated HaCaT cells were attenuated by treatment with FSCP for 24 h (a, b). Upregulation of IL-1*β* and IL-8 mRNA in TNF-*α*-treated HaCaT cells was decreased by treatment with FSCP for 24 h (c, d). Results are presented as the means ± SD of three independent experiments. ^#^*p* < 0.05 versus the control and ^∗^*p* < 0.05, ^∗∗^*p* < 0.01, and ^∗∗∗^*p* < 0.001 versus the CoCl_2_/TNF-*α*-treated group.

To examine the effects of FSCP treatment on CoCl_2_-induced expression of the key proinflammatory cytokines TNF-*α*, IL-1*β*, IL-8, and iNOS, HaCaT cells were exposed to 100 *μ*M CoCl_2_ or 20 ng/mL TNF-*α* and harvested after 12 or 24 h for RT-PCR analysis. TNF-*α* mRNA expression in HaCaT cells was dramatically upregulated by 66.2% compared to that in the control following CoCl_2_ treatment (Figure 3(a)). However, the elevated TNF-*α* mRNA levels were dramatically attenuated by treatment with 0.2, 0.5, and 1 mg/mL FSCP for 24 h in a dose-dependent manner by 49.3, 54.9, and 57.6%, respectively, compared to those of the CoCl_2_-treated group (Figure 3(a)).

iNOS mRNA showed 57.1% upregulation after stimulation with 100 *μ*M CoCl_2_ for 24 h in HaCaT cells (Figure 3(b)). Treatment of HaCaT cells with 0.2, 0.5, and 1 mg/mL FSCP for 24 h significantly downregulated iNOS mRNA levels in a dose-dependent manner, corresponding to 32.5, 37.4, and 57.0% decreases, respectively, versus the CoCl_2_-treated group (Figure 3(b)). Together, the above results indicate that FSCP are potent inhibitors of CoCl_2_-mediated proinflammatory responses and act by downregulating the transcriptional expression of TNF-*α* and iNOS in HaCaT cells.

IL-1*β* mRNA levels showed 142.0% upregulation following TNF-*α* treatment compared to the control (Figure 3(c)). However, the elevated IL-1*β* mRNA levels significantly decreased by 8.6, 49.1, and 74.5% after treatment with 1 mg/mL FSCP for 3, 12, or 24 h in a time-dependent manner, respectively, when compared to those of the TNF-*α*-treated group (Figure 3(c)). IL-8 mRNA was 282.5% upregulated in HaCaT cells after stimulation with 20 ng/mL TNF-*α* for 12 h versus the control (Figure 3(d)). After treatment with 1 mg/mL FSCP for 3, 12, or 24 h, the expression of IL-8 mRNA in HaCaT cells was downregulated by 29.3, 26.0, and 96.3% compared to that in the TNF-*α*-treated group, respectively (Figure 3(d)). Taken together, these results indicate that FSCP are potent inhibitors of CoCl_2_/TNF-*α*-mediated proinflammatory responses in HaCaT cells and act by inhibiting transcriptional expression of TNF-*α*, IL-1*β*, IL-8, and iNOS.

### 3.5. FSCP Ameliorate CoCl_2_-Induced Altered Expression of Apoptosis-Related Proteins

**Figure 4:**
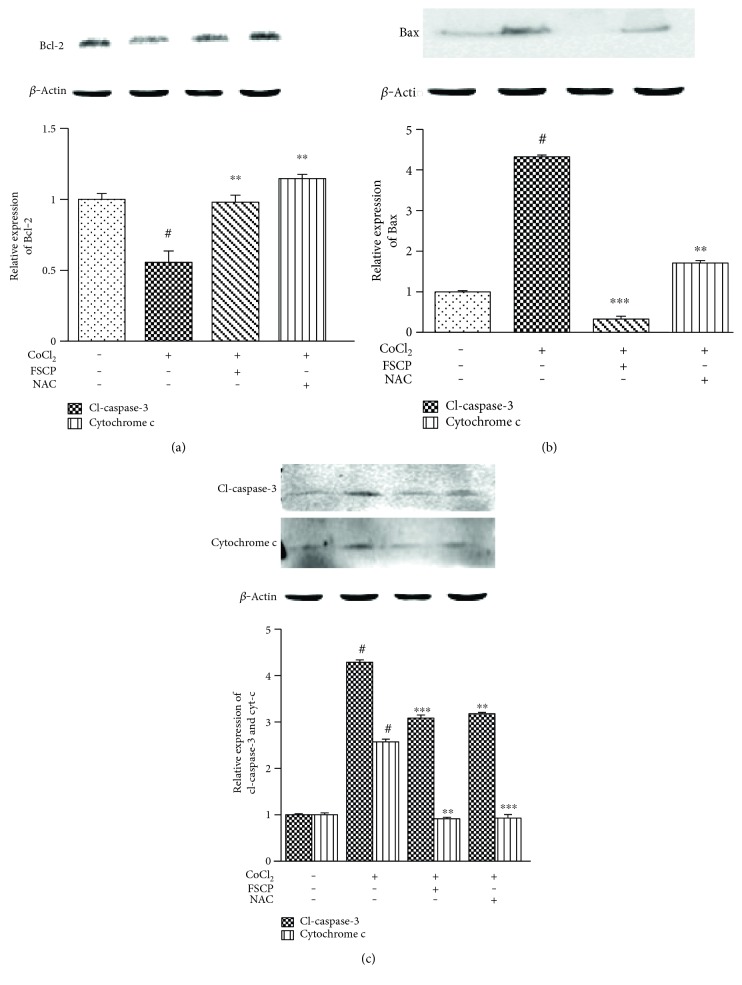
Effects of FSCP on CoCl_2_-induced altered expression of apoptosis-related proteins. The expression of apoptosis-related proteins was analyzed by western blotting. Bcl-2 downregulation and Bax upregulation in CoCl_2_-treated HaCaT cells were reversed by treatment with FSCP and NAC (a, b). Potentiated levels of caspase-3 activity and cytochrome c release in CoCl_2_-treated HaCaT cells were attenuated by treatment with FSCP and NAC (c). Results are presented as the means ± SD of three independent experiments. Cl-caspase-3: cleaved-caspase-3. ^#^*p* < 0.05 versus the control and ^∗∗^*p* < 0.01 and ^∗∗∗^*p* < 0.001 versus the CoCl_2_-treated group.

Western blot analysis showed that HaCaT cells exposed to CoCl_2_ (100 *μ*M) for 24 h had significantly lower Bcl-2 protein levels (44.5%) and higher Bax protein levels (332.6%) compared to the controls (Figures 4(a) and 4(b)). In addition, our experimental results showed that treatment with CoCl_2_ (100 *μ*M) for 24 h triggered higher release of cytochrome c (328.6%) and caspase-3 activation (157.0%) in HaCaT cells compared to the control (Figure 4(c)). However, CoCl_2_ (100 *μ*M)-induced decrease in Bcl-2 expression in HaCaT cells was completely restored to levels similar to that of the control by treatment with FSCP (1 mg/mL) or NAC (Figures 4(a)–4(c)). In addition, the enhanced induction of Bax expression by CoCl_2_ (100 *μ*M) treatment was attenuated by FSCP (1 mg/mL) or NAC treatment in HaCaT cells by 92.4 and 60.5% compared to the CoCl_2_-treated group, respectively (Figure 4(b)). Furthermore, the CoCl_2_-induced increase in cleaved caspase-3 and cytochrome c expression in HaCaT cells was significantly reduced by 28.2 and 64.5%, respectively, upon treatment with 1 mg/mL FSCP for 24 h, compared to the CoCl_2_-treated group. Similarly, treatment with NAC for 2 h decreased cleaved caspase-3 levels by 26.0% and cytochrome c expression by 64.0% (Figure 4(c)). These data suggest that FSCP confer resistance to CoCl_2_-induced apoptosis via positive regulation of Bcl-2 expression and negative control of Bax, cytochrome c, and caspase-3 in HaCaT cells.

### 3.6. FSCP Inhibit the p38/MAPK Pathway, Which Mediates CoCl_2_-Induced Injury and Oxidative Stress in HaCaT Cells

**Figure 5:**
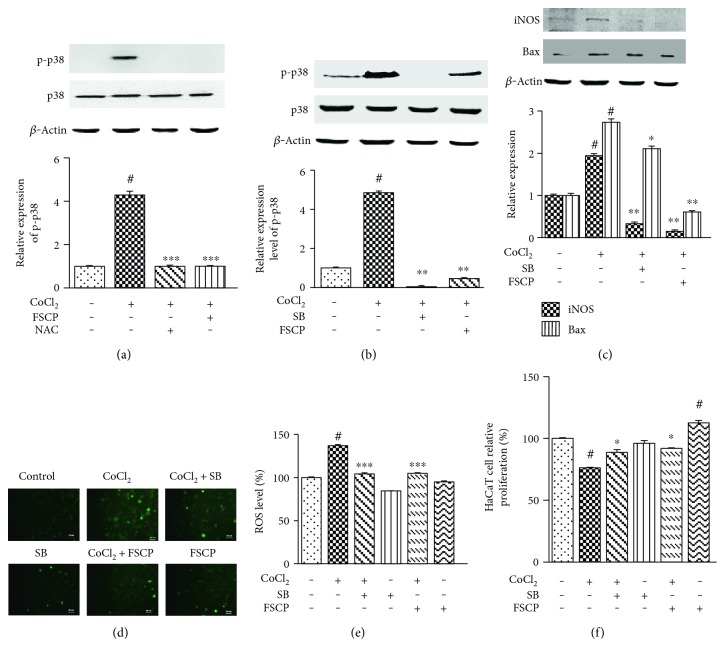
Inhibitory effects of FSCP on CoCl_2_-induced activation of p38/MAPK signaling pathway. P-p38/MAPK levels were increased in CoCl_2_-treated HaCaT cells (a). Treatment of HaCaT cells with FSCP and NAC (a) and SB203580 (SB) (b) blocked CoCl_2_-induced phosphorylation of p38/MAPK. Treatment of HaCaT cells with FSCP and SB203580 (SB) blocked CoCl_2_-induced intracellular ROS activity (c), and staining intensities were measured using ImageJ software (d). Treatment with FSCP blocked CoCl_2_-induced cytotoxicity in HaCaT cells (e). Western blot analysis showed that the treatment of HaCaT cells with FSCP and SB203580 (SB) suppressed CoCl_2_-induced elevated expression of iNOS and Bax (f). Results are presented as the means ± SD of three independent experiments. ^#^*p* < 0.05 versus the control and ^∗^*p* < 0.05, ^∗∗^*p* < 0.01, and ^∗∗∗^*p* < 0.001 versus the CoCl_2_-treated group.

As shown in Figures 5(a) and 5(b), treatment of HaCaT cells with 100 *μ*M CoCl_2_ for 24 h resulted in the marked upregulation of p-p38/MAPK compared to the control group (*p* < 0.05). However, total p38/MAPK levels were not altered. To determine the exact role of p38/MAPK in CoCl_2_-induced injury, we tested the effect of the kinase inhibitor on CoCl_2_-induced expression of p-p38/MAPK. Cotreatment of cells with FSCP (1 mg/mL) and CoCl_2_ for 24 h or treatment with NAC for 2 h completely inhibited CoCl_2_-induced p38/MAPK phosphorylation (Figure 5(a)). Subsequently, HaCaT cells were treated with 100 *μ*M CoCl_2_ for 24 h after pretreatment with 20 *μ*M SB203580 (a selective inhibitor of p38/MAPK) for 1 h. As shown in Figure 5(b), treatment of HaCaT cells with SB203580 (20 *μ*M) completely blocked CoCl_2_-induced phosphorylation of p38/MAPK.

To investigate whether p38/MAPK activation is involved in the observed CoCl_2_-induced increase in iNOS and Bax expression in HaCaT cells, we examined the effect of p38/MAPK inhibition on CoCl_2_-induced expression of iNOS and Bax. Western blot analysis showed that treatment of cells with CoCl_2_ for 24 h markedly enhanced iNOS and Bax protein expression by 94.2 and 173.4%, respectively, compared to the control group (*p* < 0.05) (Figure 5(c)). However, both pretreatment with 20 *μ*M SB203580 for 1 h followed by CoCl_2_ treatment and combined treatment with 1 mg/mL FSCP and CoCl_2_ for 24 h effectively blocked CoCl_2_-induced upregulation of iNOS and Bax (Figure 5(c)).

To determine the role of the p38/MAPK pathway in CoCl_2_-induced ROS production, HaCaT cells were pretreated with 20 *μ*M SB203580 for 1 h before exposure to 100 *μ*M CoCl_2_ for 24 h. Cells were then subjected to DCFH-DA staining and photofluorography to determine the levels of intracellular ROS. As shown in Figures 5(d) and 5(e), pretreatment of HaCaT cells with SB203580 prior to CoCl_2_ treatment led to a significant decrease in DCF-derived fluorescence comparable to the control group, demonstrating complete suppression of CoCl_2_-induced intracellular ROS production. On the other hand, treatment with SB203580 alone had no significant effect on cellular ROS activity in HaCaT cells. These findings suggest that the p38/MAPK pathway is involved in CoCl_2_-induced ROS generation. Similarly, treatment with 1 mg/mL FSCP significantly attenuated CoCl_2_-induced intracellular ROS generation to levels comparable to that of the control group, whereas treatment with FSCP alone had no effect on ROS activity in HaCaT cells (Figures 5(d) and 5(e)).

Furthermore, pretreatment of cells with 20 *μ*M SB203580 for 1 h before exposure to CoCl_2_ effectively blocked the CoCl_2_-induced cytotoxicity (*p* < 0.01), whereas SB203580 alone did not affect cell viability in HaCaT cells (Figure 5(f)). Similarly, treatment with 1 mg/mL FSCP also significantly ameliorated CoCl_2_-induced cytotoxicity, whereas treatment with 1 mg/mL FSCP alone stimulated cell proliferation in HaCaT cells (Figure 5(f)).

The above results indicate that FSCP inhibit the p38/MAPK pathway which mediates CoCl_2_-induced cytotoxicity, oxidative stress, and upregulation of iNOS and Bax protein levels in HaCaT cells.

### 3.7. FSCP Inhibit TNF-*α*-Induced Activation of MAPK Pathway in HaCaT Cells

**Figure 6:**
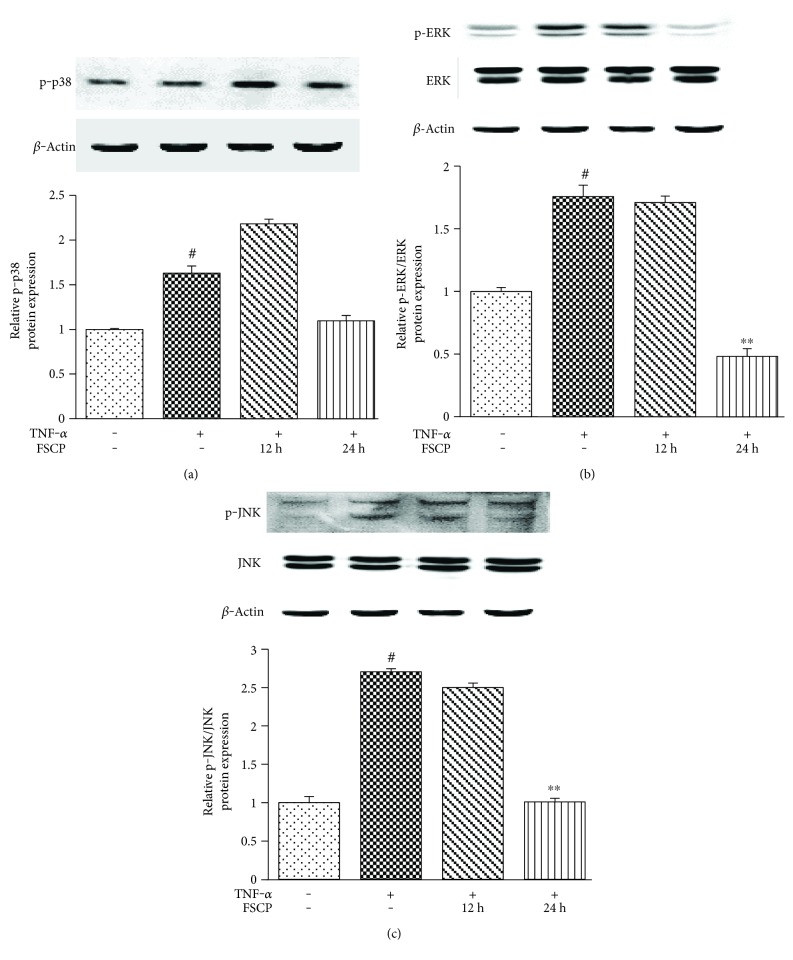
Inhibitory effects of FSCP on TNF-*α*-induced activation of the MAPK signaling pathway. Treatment of HaCaT cells with FSCP did not significantly inhibit TNF-*α*-induced increase in p38/MAPK phosphorylation (a). Treatment of HaCaT cells with FSCP suppressed TNF-*α*-induced ERK (b) and JNK (c) activation. Results are presented as the means ± SD of three independent experiments. ^#^*p* < 0.05 versus the control and ^∗∗^*p* < 0.01 versus the TNF-*α*-treated group.

We performed western blot analysis to evaluate the effects of FSCP on TNF-*α*-induced MAPK activation in HaCaT cells. As shown in Figure 6(a), HaCaT cells treated with 20 ng/mL TNF-*α* for 12 h showed 63.0% upregulation of p-p38/MAPK compared to the control group. However, pretreatment of cells with FSCP (1 mg/mL) for 12 or 24 h prior to TNF-*α* treatment did not significantly inhibit TNF-*α*-induced p38/MAPK phosphorylation (Figure 6(a)). As shown in Figure 6(b), the treatment of HaCaT cells with 20 ng/mL TNF-*α* for 12 h led to a remarkable increase (75.7%) in the protein levels of p-ERK compared to the control group, whereas the total ERK protein levels were not altered. Notably, TNF-*α*-induced ERK activation was substantially suppressed (72.5%) after pretreatment of HaCaT cells with 1 mg/mL FSCP for 24 h compared to the TNF-*α*-treated group (Figure 6(b)). Consistent with this result, treatment of HaCaT cells with 20 ng/mL TNF-*α* for 12 h induced a remarkable increase (170.5%) in protein levels of p-JNK compared to the control group, whereas total protein levels of JNK were not altered. Similar to ERK, TNF-*α*-induced JNK activation was significantly reduced (62.7%) upon pretreatment of HaCaT cells with 1 mg/mL FSCP for 24 h compared to the TNF-*α*-treated group (Figure 6(c)).

### 3.8. FSCP Inhibit TNF-*α*-Induced NF-*κ*B Activation

**Figure 7:**
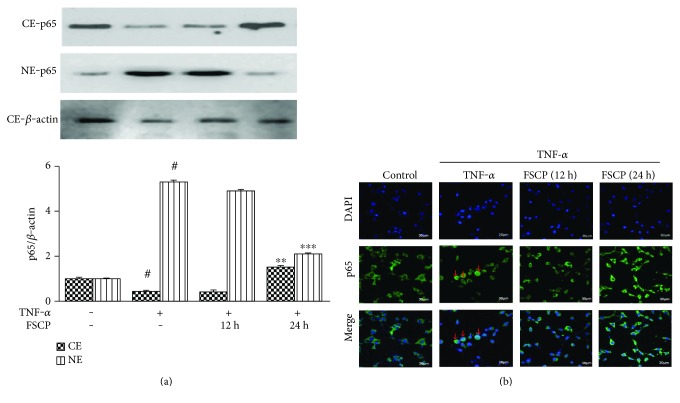
Inhibitory effects of FSCP on TNF-*α*-induced activation of the NF-*κ*B signaling pathway. Western blot analysis showed NF-*κ*B p65 levels were decreased in the cytoplasm and increased in the nucleus in TNF-*α*-treated HaCaT cells (a). Immunofluorescence microscopic assay revealed that FSCP prevented the translocation of NF-*κ*B p65 to the nucleus in TNF-*α*-treated HaCaT cells (b). Results are presented as the means ± SD of three independent experiments. CE: cytoplasmic extracts; NE: nuclear extract. ^#^*p* < 0.05 versus the control and ^∗∗^*p* < 0.01 and ^∗∗∗^*p* < 0.001 versus the TNF-*α*-treated group.

**Figure 8:**
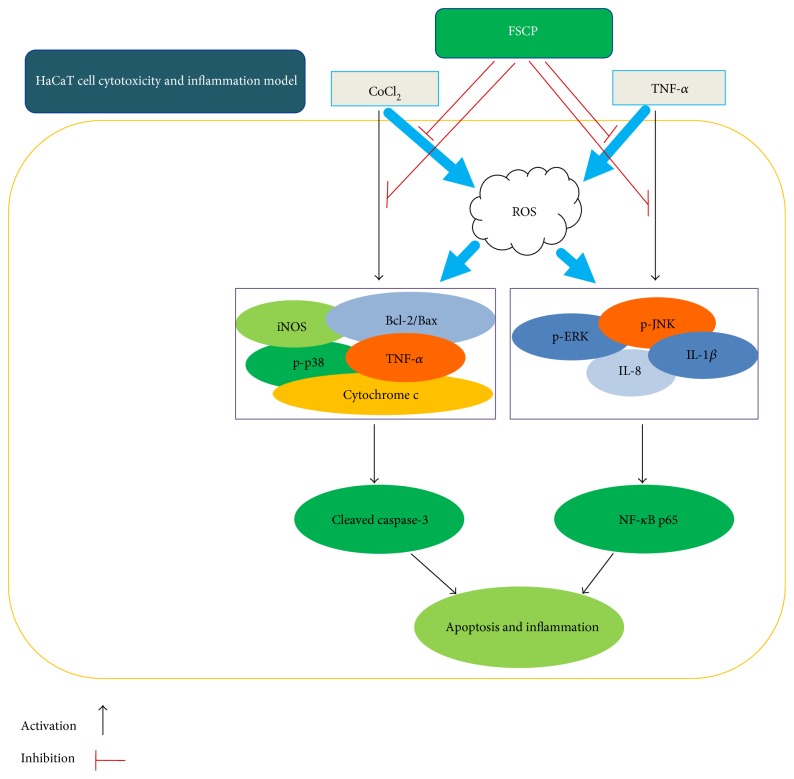
Schematic representation of the regulatory mechanism of FSCP on CoCl_2_-/TNF-*α*-induced oxidative and inflammatory stress in HaCaT cells.

To further investigate the mechanisms by which FSCP inhibit proinflammatory responses, we investigated whether FSCP can prevent nuclear translocation of the p65 subunit of NF-*κ*B. Western blot analysis (Figure 7(a)) showed that following exposure to 20 ng/mL TNF-*α*, NF-*κ*B p65 levels were 56.7% lower in the cytoplasm but were 430.0% higher in the nucleus compared to the control group. However, pretreatment with 1 mg/mL FSCP for 24 h remarkably increased TNF-*α*-induced p65 levels in the cytoplasmic fractions by 250.0% but reduced p65 levels in nuclear fractions by 60.4% compared to the TNF-*α*-treated cells. Furthermore, we confirmed the inhibition of TNF-*α*-induced NF-*κ*B activation by performing an immunofluorescence microscopy assay. As shown in Figure 8(b), FSCP treatment prevented nuclear translocation of the p65 subunit of NF-*κ*B, consistent with the results obtained from the immunoblot assay. These results suggest that FSCP inhibit NF-*κ*B activation in HaCaT cells by preventing nuclear translocation of NF-*κ*B.

## 4. Discussion and Conclusion

The present study is the first to investigate the effects of FSCP on CoCl_2_- and TNF-*α*-induced oxidative and inflammatory stress responses in HaCaT cells. We also provide molecular evidence demonstrating that FSCP exert antioxidative effects and provide protection against skin damage due to various stimuli, including ultraviolet radiation from sunlight, which causes oxidative stress. FSCP can also serve as a potential anti-inflammatory drug for the treatment of several inflammatory skin conditions, such as sunburn, eczema, and psoriasis. Inflammatory, allergic, and autoimmune skin diseases such as atopic dermatitis, urticaria, and psoriasis can also be mediated by oxidative stress [26]. We hypothesized that FSCP exert protective effects against oxidative stress- and inflammation-induced skin injury.

Many studies have investigated the biological functions of fish collagen peptides [27]. However, the biological effects of fish collagen peptides on keratinocytes have received surprisingly little scientific interest. The antioxidant activities of peptides obtained from the hydrolysates of collagen or gelatin from different marine species have been documented. Mendis et al. [28] demonstrated that gelatin hydrolysates obtained from jumbo squid (*Dosidicus gigas*) skin, particularly those with molecular masses ranging from 0.8 to 1.3 kDa, exhibit strong antioxidant activity and enhance the viability of human lung fibroblasts damaged by free radicals and oxidation. Kim et al. [29] showed that purified peptides from gelatin hydrolysates prepared from Alaska pollock (*Gadus chalcogrammus*) skin (ranging from 1.5 to 4.5 kDa) exert potent antioxidative activity against oxidant injury caused by *tert*-butyl hydroperoxide in Donryu rat liver cells. Ngo et al. [30] showed that gelatin hydrolysates from the skin of Pacific cod (*Gadus macrocephalus*) contain free radical-scavenging peptides (ranging from 0.3 to 0.5 kDa) that were shown to exert protective effects against oxidation-induced DNA damage in RAW264.7 mouse macrophages. Likewise, Himaya et al. [31] observed that gelatin hydrolysates derived from the skin of the Pacific cod (*Gadus macrocephalus*) contain small peptides that function as efficient scavengers of intracellular ROS in RAW264.7 cells. The above findings agree with our present results demonstrating that FSCP from tilapia exert potent antioxidative effects on cultured human keratinocytes.

Synthetic antioxidants have been well recognized for their use in the treatment of diabetes mellitus and other related diseases that are characterized by excessive ROS generation; however, studies have shown that synthetic antioxidants are unsafe [32]. In the past few decades, concerns have been raised regarding the potential adverse effects of synthetic antioxidants on health, such as carcinogenicity [33]. Thus, research efforts have been focused on the development and utilization of natural antioxidants. Most natural antioxidants that have been identified so far are present in almost all plants, microorganisms, fungi, and even animal tissues. Recently, peptides derived from marine sources have particularly attracted interest owing to their broad-spectrum bioactivities. Despite the potential importance of marine peptides in human health, many bioactive marine peptides, as well as their biological functions, remain to be elucidated. Determining the bioregulatory roles of marine peptides and their corresponding mechanisms of action will provide valuable information and serve as the basis for developing new therapeutic strategies for the treatment of many intractable diseases.

ROS alter the DNA by inducing mutations, deletions, gene amplification, and rearrangements [34]. These changes can trigger the dysregulation of proapoptotic or antiapoptotic pathways, leading to apoptotic cell death. Our results demonstrated that FSCP act as potent suppressors of CoCl_2_-induced cell apoptosis in HaCaT cells by upregulating Bcl-2 expression and inhibiting Bax expression, cytochrome c release, and caspase-3 activation. Consistent with our findings, modulation of apoptotic signaling effectors (Bcl-2, Bax, caspase-3 cleavage, and cytochrome c) in CoCl_2_-treated skin keratinocytes has been reported [35]. Several studies also revealed that CoCl_2_ exerts its cytotoxic effects by inducing ROS production and cellular damage [11]. Taken together, these findings indicate that the amelioration of CoCl_2_- or TNF-*α*-induced cytotoxicity in HaCaT cells is mediated by the antioxidant and antiapoptotic properties of FSCP. Detailed understanding of the mechanisms by which FSCP mediate the repair of cell injuries induced by oxidative stress and activation of apoptotic cell death pathway in human keratinocytes would highlight their promising use in the treatment of many diseases caused by excessive ROS generation and perturbations in apoptotic balance.

In this study, the molecular mass distribution of the obtained FSCP ranged from 0.1 to 1.3 kDa based on TripleTOF MS analysis. In general, hydrolyzed collagen peptides derived from marine sources, such as fish scales, have molecular weights ranging from 1 to 5 kDa and have amino acid distributions similar to that of their corresponding native collagen peptide [36]. Recently, marine collagen peptides from chum salmon with molecular weights between 0.1 and 0.9 kDa were shown to facilitate wounding healing and promote the physiological and neurobehavioral development of male rats with perinatal asphyxia [37, 38]. Hydrolyzed collagen peptides from tilapia fish scales with molecular weights ranging from 0.7 to 1.3 kDa can induce multidirectional differentiation of rat bone marrow mesenchymal stem cells and osteogenic differentiation of human periodontal ligament cells and modulate the behavior of macrophages [14, 22]. Previous studies have suggested that low molecular weight peptides from animal or marine sources act by exposing more active sites in proteins and thus more effectively regulate cell growth than larger peptides [39].

The amino acid composition of fish collagen is similar to that of mammalian collagen but contains less proline and hydroxyproline and more serine and threonine residues, particularly in fish collagen derived from cold water species [40]. These differences in amino acid composition, especially in terms of hydroxyproline content, are responsible for differences in collagen properties, such as rigidity, temperature stability, and denaturation temperature [41]. On the other hand, collagen derived from warm water fish species, including tilapia, can exhibit similar amino acid composition, rheological properties, and thermostability to that of mammalian collagen [42], suggesting that tilapia collagen can be used as an alternative to mammalian collagen in biomedical applications.

The present study demonstrated that FSCP can promote proliferation in HaCaT cells. Consistent with our findings, Liu and Sun [22] also reported that tilapia-derived FSCP with molecular weights ranging from 0.7 to 1.3 kDa promoted the growth of rat bone marrow mesenchymal stem cells. In addition, results from our previous study suggested that tilapia FSCP contained in the FC/PCL nanofibrous scaffolds contribute to enhanced proliferation of mouse thymic epithelial cells [43]. Furthermore, Liu et al. [44] showed that bovine collagen peptide compounds promoted the proliferation and differentiation of MC3T3-E1 preosteoblasts. Together, the above results suggest that low molecular weight collagen peptides can promote cell proliferation, regardless of the source organism.

To validate whether FSCP exhibit anti-inflammatory effects on human keratinocytes, we measured the expression levels of critical proinflammatory cytokines, namely, TNF-*α*, IL-1*β*, IL-8, and iNOS, in CoCl_2_- or TNF-*α*-treated HaCaT cells. Cytokines modulate the intricate cell-to-cell communication networks and thus play a crucial role in orchestrating host defense, including inflammatory and immune responses; cytokines act by regulating cell proliferation, survival, growth, differentiation, migration, and activation [45]. In addition to lymphocytes, macrophages, mast cells, and neutrophils, nonimmunological cells such as endothelial cells, keratinocytes, and fibroblasts are also capable of producing cytokines. In recent years, the role of cytokines in many immune-related diseases, including skin diseases, has been extensively studied to obtain a detailed understanding of pathophysiologic processes underlying these disorders. Currently, cytokine and anticytokine therapies are increasingly used for the treatment of various diseases, including skin diseases. Furthermore, the diagnostic determination of cytokine expression has now been adopted for clinical use. Thus, detailed investigation of the complex action of these cytokines, including their proinflammatory and immunosuppressive properties, has become crucial [46]. In the present study, we found that FSCP exert potent anti-inflammatory effects on human keratinocytes by inhibiting the expression of the key proinflammatory cytokines. These results suggest that FSCP can serve as novel therapeutic agents that can be used for the treatment of inflammatory and immunologic skin diseases. These results are consistent with those of a previous study demonstrating that fucosterol, a natural product, attenuated CoCl_2_-induced upregulation of inflammatory molecules, such as TNF-*α*, IL-1*β*, and IL-6, in HaCaT cells [47].

MAPK signal transduction pathways are involved in a wide range of fundamental cellular processes, including cell growth, differentiation, survival, apoptosis, migration, inflammation, and response to environmental stresses [48]. In particular, p38/MAPK signaling plays a critical role in the modulation of immune-mediated inflammatory responses, including cellular and humoral autoimmune responses, and has been linked to several autoimmune diseases [49]. The p38/MAPK pathway is also essential for cellular survival, proliferation, differentiation, and apoptosis [50]. Our results showed that treatment of HaCaT cells with CoCl_2_ induced activation of p38/MAPK signaling, but p38/MAPK activation was abolished by treatment with SB203580 or cotreatment with FSCP and NAC, suggesting that CoCl_2_-induced oxidative stress injury in HaCaT cells is mediated by p38/MAPK. Similar to the mode of action of NAC, FSCP ameliorate CoCl_2_-induced oxidative stress in HaCaT cells by blocking p38/MAPK activation. In addition, treatment of HaCaT cells with CoCl_2_ resulted in decreased cell viability, increased Bax protein levels, and induction of iNOS expression; these CoCl_2_-induced cytotoxic and inflammatory responses were significantly blocked by both SB203580 and FSCP. Together, these data indicate that FSCP play a protective role in the amelioration of the oxidative stress-induced cytotoxic and inflammatory responses in human keratinocytes.

The p38/MAPK signaling pathway also participates in chronic inflammatory skin pathologies such as skin inflammation and epidermal cell apoptosis, which are triggered by various stimuli like ultraviolet irradiation or burn wounds [51]. Aberrant p38/MAPK activation has been observed in the epidermal lesions of several immune-mediated skin diseases, including psoriasis and pemphigus vulgaris [49]; therefore, the p38/MAPK signaling pathway can serve as a therapeutic target in the treatment of these diseases [52]. The p38/MAPK signaling pathway plays a crucial role in promoting inflammatory responses by stimulating the production of inflammatory cytokines and mediators and thus has been considered a promising target for the treatment of chronic inflammatory diseases [53]. In this context, we can hypothesize that the use of FSCP, as a potent p38/MAPK inhibitor, is effective for therapeutic management of a number of inflammatory and immunological skin diseases. Our results are supported by previous studies demonstrating that p38/MAPK/iNOS is involved in apoptosis in rat PC12 pheochromocytoma cells and rat RINm5F pancreatic islet *β* cells [54, 55]. Similarly, the p38/MAPK/Bax pathway was shown to be associated with ultraviolet-damaged keratinocytes [56]. Altogether, these data suggest that FSCP protect against CoCl_2_-induced cytotoxicity in HaCaT cells via the p38/MAPK/iNOS and p38/MAPK/Bax pathways.

TNF-*α* acts as a master switch for activating inflammatory responses and innate immunity processes by activating multiple downstream pathways, including the MAPK, NF-*κ*B, and death signaling pathways. The induction of many proinflammatory cytokines and immune-regulatory proteins is mainly mediated by the NF-*κ*B or MAPK pathway [57]. TNF-*α* plays important roles in the pathology of inflammatory skin diseases, such as psoriasis and atopic dermatitis [58]. In general, ERKs are activated by mitogens and differentiation signals, while the JNK and p38 MAPK pathways are activated by stress stimuli. TNF-*α* can activate the MAPK, p38/MAPK, ERK, and JNK pathways [59]. In addition, the NF-*κ*B pathway, a prototypical proinflammatory signaling pathway which is primarily activated by proinflammatory cytokines, such as TNF-*α* and IL-1, is a critical regulator of apoptosis, inflammation, and the development of autoimmune diseases [60, 61]. NF-*κ*B is also involved in the expression of other proinflammatory cytokines, as well as chemokines, and adhesion molecules. Thus, the TNF-*α*, NF-*κ*B, and MAPK pathways represent potential therapeutic targets for the development of anti-inflammatory drugs. Our study showed that FSCP suppress the ERK and JNK activation via TNF-*α*; however, p38/MAPK phosphorylation was shown to be unaffected by treatment with FSCP. Furthermore, we demonstrated that FSCP attenuate the expression of TNF-*α*-induced IL-1*β* and IL-8, as well as ERK, JNK, and NF-*κ*B activation in HaCaT cells. Thus, FSCP-induced downregulation of IL-1*β* and IL-8 in TNF-*α*-treated HaCaT cells appears to be mediated by the combined inhibitory mechanisms of multiple signaling pathways, including NF-*κ*B, ERK, and JNK.

Furthermore, it has recently been found that collagen-derived peptides have mineral binding activity. Guo et al. [62] identified Alaska pollock fish skin collagen-derived Ca, Fe, and Cu chelating peptides with molecular weights mainly ranging from 0.5 to 2 kDa, indicating FSCP as a good source of peptides with potential applications as functional ingredients in the management of mineral deficiencies. In addition, it was demonstrated that collagen peptides, from scales of four kinds of fish (*Lates calcarifer*, *Mugil cephalus*, *Chanos chanos*, and *Oreochromis* spp.) with molecular weights averaged 1.3 kDa, exhibit Fe(II)-binding activity, indicating that FSCP could be applied in industry as a bioresource [63]. These findings suggest that FSCP used in this study might have metal chelating activity, providing an insight into a possible additional mechanism of action for tilapia-derived FSCP in the protection from CoCl_2_-induced HaCaT cell injury.

In conclusion, the findings of the present study demonstrate for the first time that FSCP stimulate proliferation, ameliorate oxidative injury, and inhibit the expression of key proinflammatory cytokines (TNF-*α*, IL-1*β*, IL-8, and iNOS) in CoCl_2_- or TNF-*α*-stimulated HaCaT cells (Figure 8). In addition, the inhibitory effects of FSCP on cytotoxicity and the induction of proinflammatory cytokine expression are likely to be associated with the suppression of the ROS, MAPK (p38/MAPK, ERK, and JNK), and NF-*κ*B signaling pathways (Figure 8). Therefore, our data suggest that FSCP are promising immunomodulatory agents that can be used for the treatment of inflammatory or immune-mediated skin diseases. Furthermore, our results provide new insights into the molecular mechanisms of FSCP for their potential therapeutic use in the prevention and treatment of a variety of the oxidative- or inflammatory stress-related inflammation and injuries.

## Supplementary Material

 Supplementary Figure 1: TripleTOF MS/MS spectra of FSCP extracted from tilapia. The molecular mass distribution was determined. Mass spectra were recorded between m/z 100 and 1500. Supplementary Table 1 Amino acid composition of FSCP.

## Figures and Tables

**Table 1:** RT-PCR primer names and their sequences.

Target gene	Forward primer sequence	Reverse primer sequence
iNOS	GGTGGAAGCAGTAACAAAGGA	GACCTGATGTTGCCGTTGTTG
TNF-*α*	CTGCTGCACTTTGGAGTGAT	AGATGATCTGACTGCCTGGG
IL-8	ACATGACTTCCAAGCTGGCCG	TTTATGAATTCTCAGCCCTC
IL-1*β*	AAAAGCTTGGTGATGTCTGG	TTTCAACACGCAGGACAGG
GAPDH	GAAGGTGAAGGTCGGAGT	GAAGATGGTGATGGGATTTC
