# Supplementary material for: Fish Scale Collagen Peptides Protect against CoCl2/TNF-α-Induced Cytotoxicity and Inflammation via Inhibition of ROS, MAPK, and NF-κB Pathways in HaCaT Cells
Source: Oxid Med Cell Longev. 2017 Jun 22;2017:9703609. doi: 10.1155/2017/9703609 (PMC5498912; doi:10.1155/2017/9703609)
Supplement: Supplementary file 1 — Supplementary Figure 1: TripleTOF MS/MS spectra of FSCP extracted from tilapia. The molecular mass distribution was determined. Mass spectra were recorded between m/z 100 and 1500. Supplementary Table 1 Amino acid composition of FSCP. [file 9703609.f1.pptx]

## Slide 1
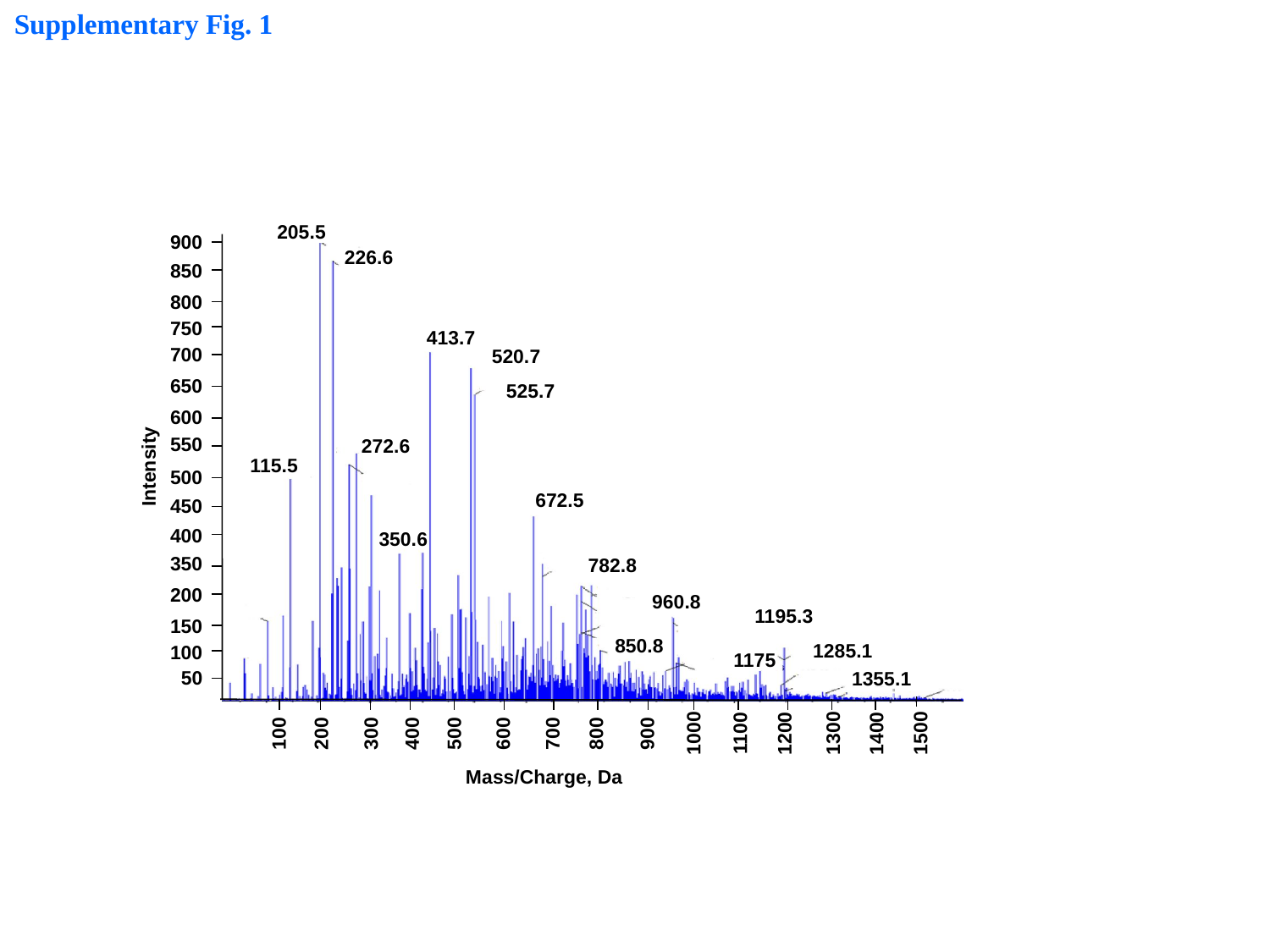

Supplementary Fig. 1
205.5
900
850
800
750
700
650
600
550
500
450
400
350
200
150
100
50
226.6
413.7
520.7
525.7
272.6
115.5
Intensity
672.5
350.6
782.8
960.8
1195.3
850.8
1285.1
1175
1355.1
100
200
300
400
500
600
700
800
900
1000
1100
1200
1300
1400
1500
Mass/Charge, Da

## Slide 2
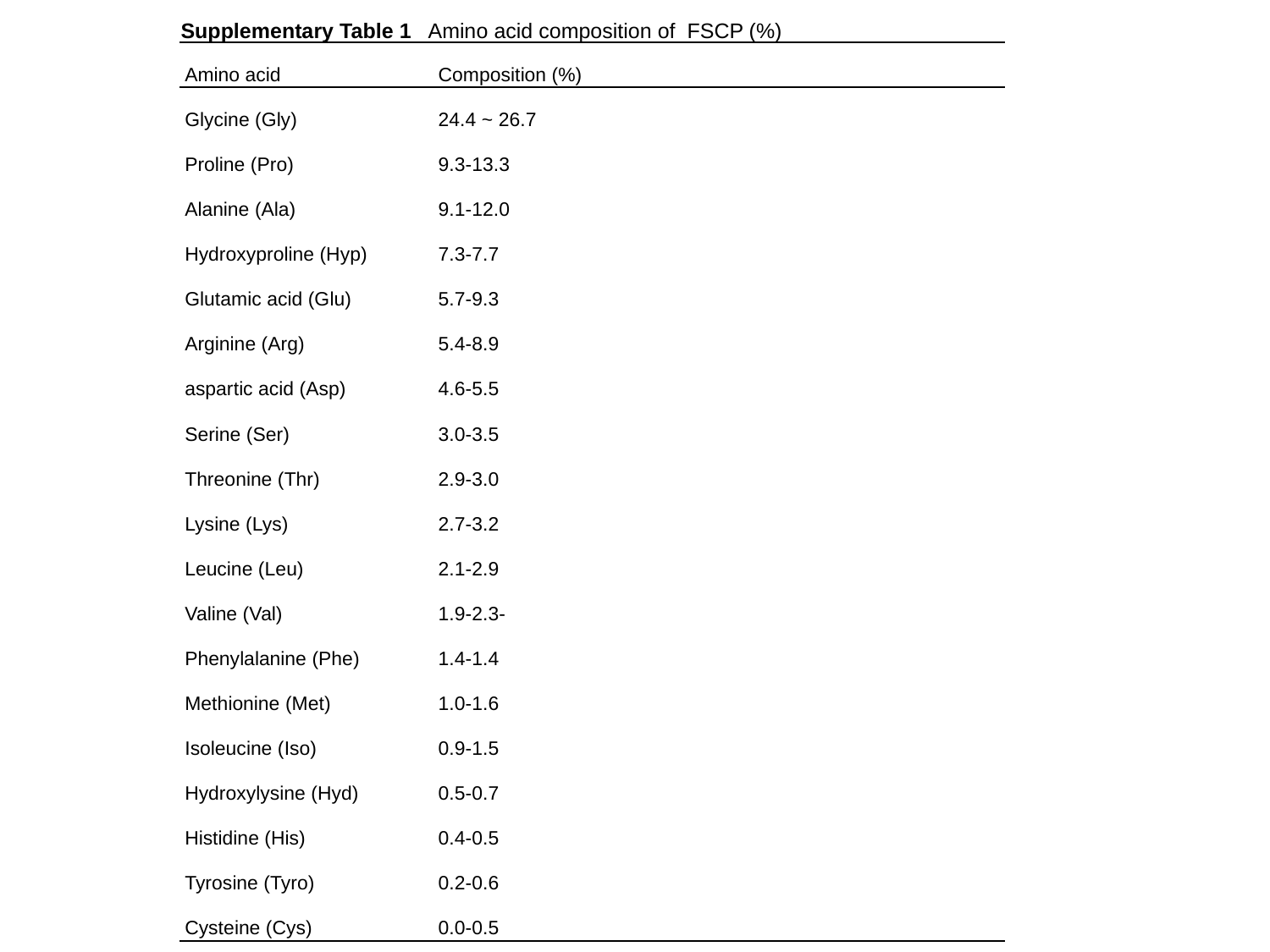

Supplementary Table 1 Amino acid composition of FSCP (%)
| Amino acid | Composition (%) |
| --- | --- |
| Glycine (Gly) | 24.4 ~ 26.7 |
| Proline (Pro) | 9.3-13.3 |
| Alanine (Ala) | 9.1-12.0 |
| Hydroxyproline (Hyp) | 7.3-7.7 |
| Glutamic acid (Glu) | 5.7-9.3 |
| Arginine (Arg) | 5.4-8.9 |
| aspartic acid (Asp) | 4.6-5.5 |
| Serine (Ser) | 3.0-3.5 |
| Threonine (Thr) | 2.9-3.0 |
| Lysine (Lys) | 2.7-3.2 |
| Leucine (Leu) | 2.1-2.9 |
| Valine (Val) | 1.9-2.3- |
| Phenylalanine (Phe) | 1.4-1.4 |
| Methionine (Met) | 1.0-1.6 |
| Isoleucine (Iso) | 0.9-1.5 |
| Hydroxylysine (Hyd) | 0.5-0.7 |
| Histidine (His) | 0.4-0.5 |
| Tyrosine (Tyro) | 0.2-0.6 |
| Cysteine (Cys) | 0.0-0.5 |
