# Supplementary figures and images for: Fish Scale Collagen Peptides Protect against CoCl2/TNF-α-Induced Cytotoxicity and Inflammation via Inhibition of ROS, MAPK, and NF-κB Pathways in HaCaT Cells
Source: Oxid Med Cell Longev. 2017 Jun 22;2017:9703609. doi: 10.1155/2017/9703609 (PMC5498912; doi:10.1155/2017/9703609)

## Slide 1
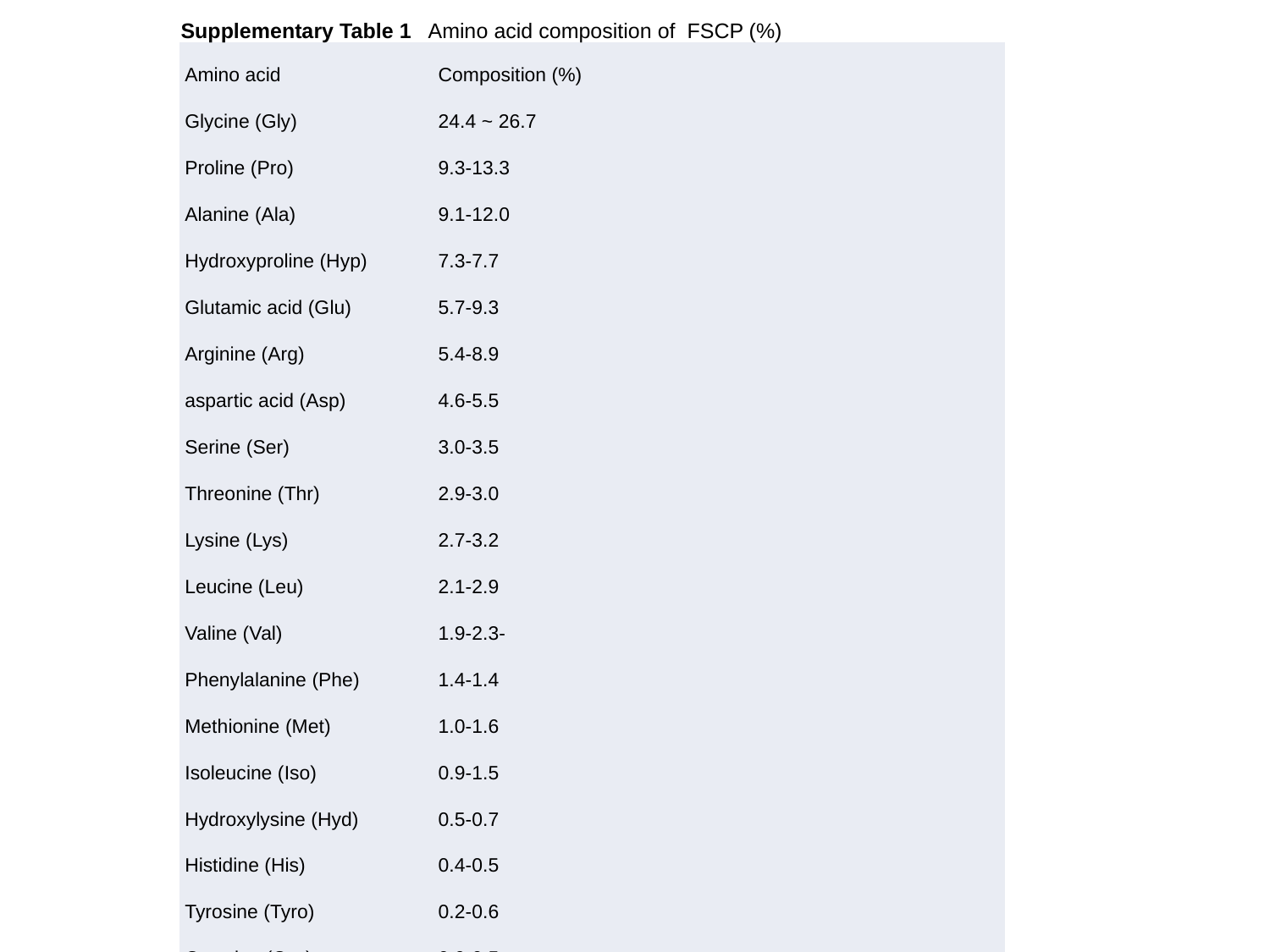

Supplement: Supplementary file 2 [file 9703609.f2.pptx]
